# Supplementary figures and images for: U2 snRNA structure is influenced by SF3A and SF3B proteins but not by SF3B inhibitors
Source: PLoS One. 2021 Oct 14;16(10):e0258551. doi: 10.1371/journal.pone.0258551 (PMC8516221; doi:10.1371/journal.pone.0258551)

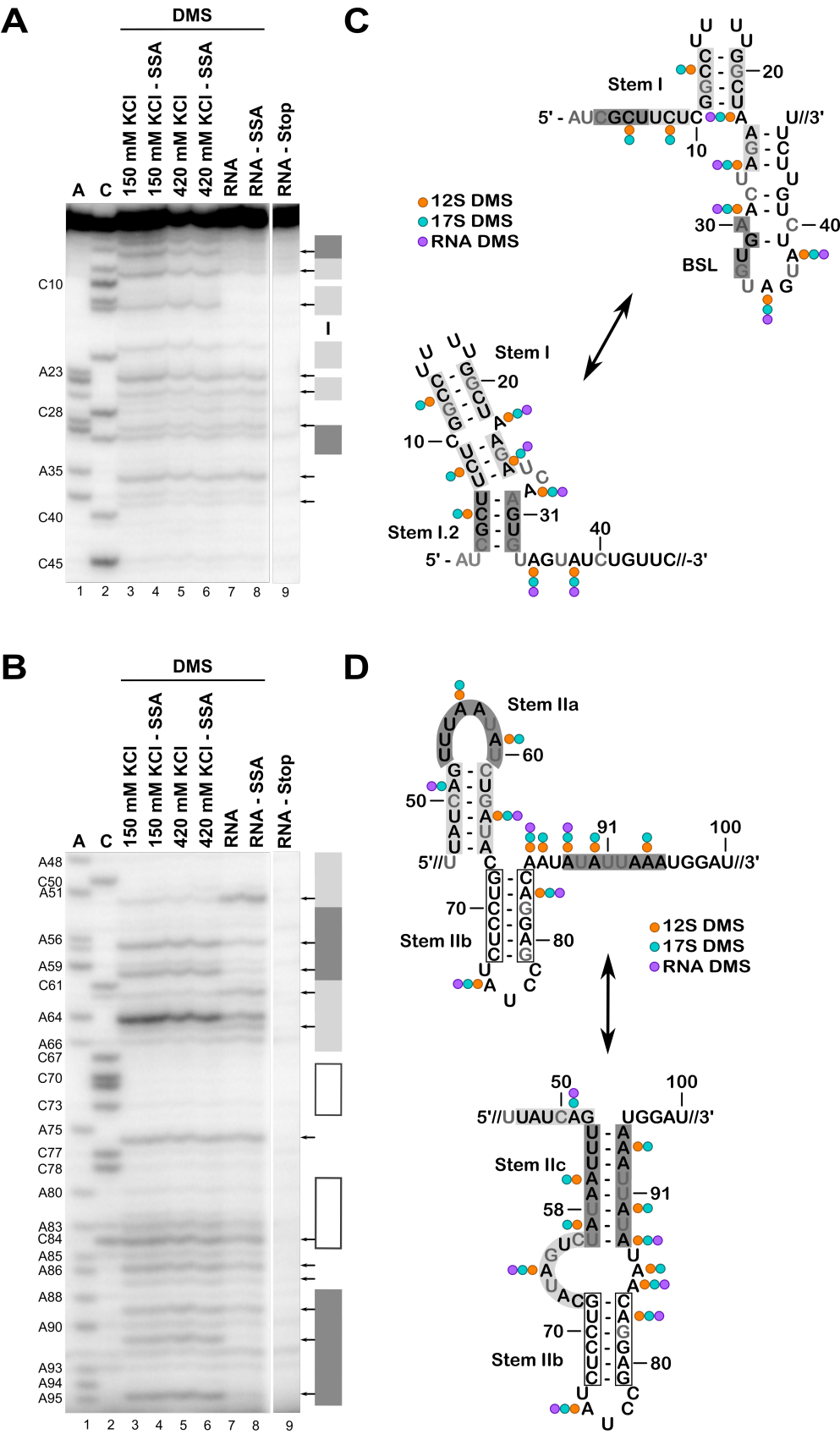

Supplement: S1 Fig — (A-B) Sequencing gel analysis of reverse transcription primer extension products from U2 snRNA isolated from nuclear extract with 17S U2 snRNP (150 mM KCl) or 12S snRNP (420 mM KCl) probed with DMS. Protein-free RNA purified from nuclear extract (RNA) was also probed. The gels are labeled like Fig 1, with lanes 9 showing a control reaction with DMSO and parallel sequencing reactions (lanes 1–2). Arrows point to primer extension products observed consistently across triplicate experiments. Nucleotides 1–46 are shown in A, and 47–95 in B. (C & D) Reactivity patterns mapped on two competing structural models of U2 snRNA with the structure most consistent with 17S U2 snRNP data shown on top. Nucleotides in grey correlate with primer extension stops in controls. The circles denote reactivity to DMS for 17S (green) or 12S (orange) U2 snRNPs or protein-free U2 snRNA (purple). (PDF) [file pone.0258551.s001.pdf]
