# Supplementary material for: U2 snRNA structure is influenced by SF3A and SF3B proteins but not by SF3B inhibitors
Source: PLoS One. 2021 Oct 14;16(10):e0258551. doi: 10.1371/journal.pone.0258551 (PMC8516221; doi:10.1371/journal.pone.0258551)

Supporting Information S2 Fig

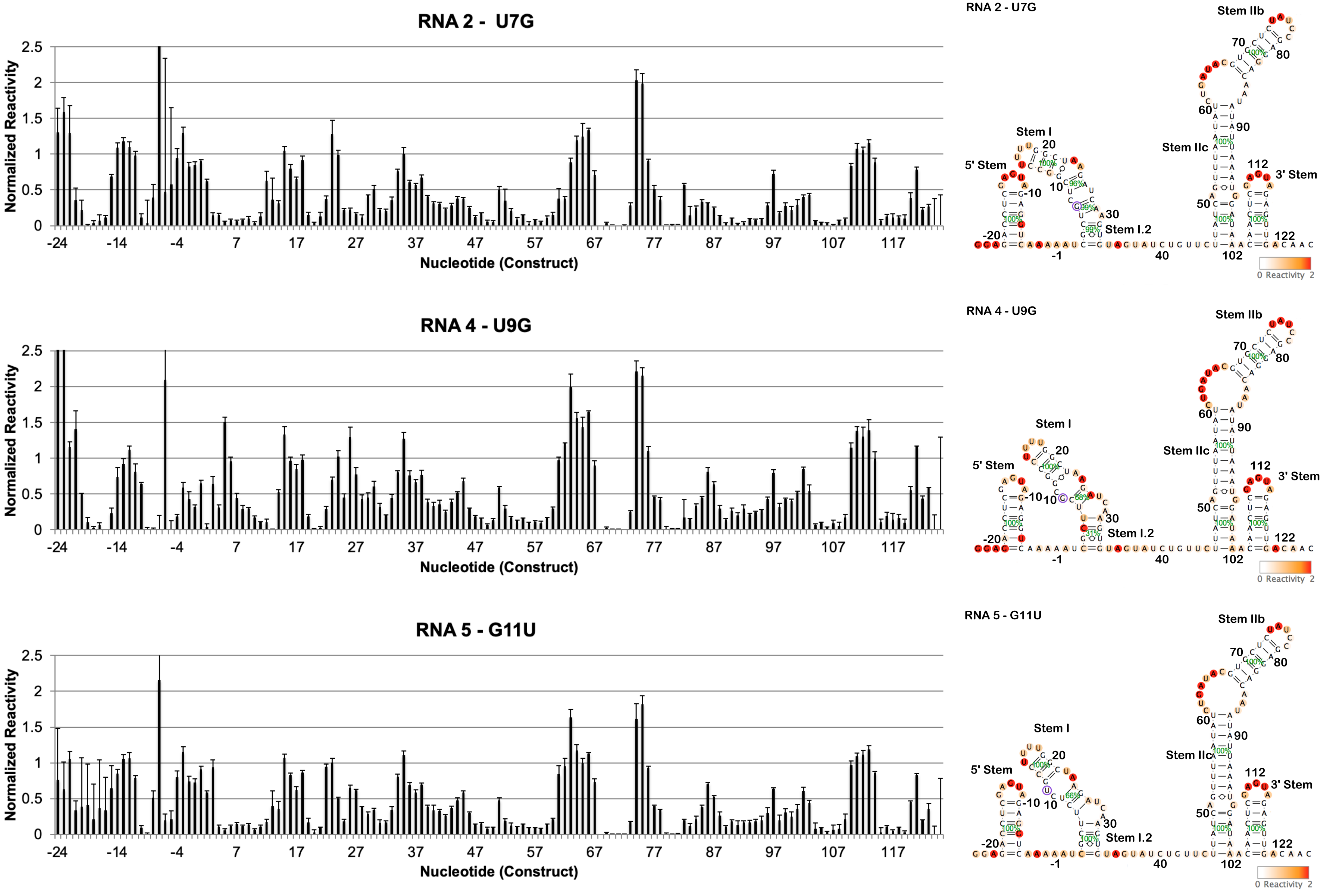

Supporting Information S2 Fig (cont)

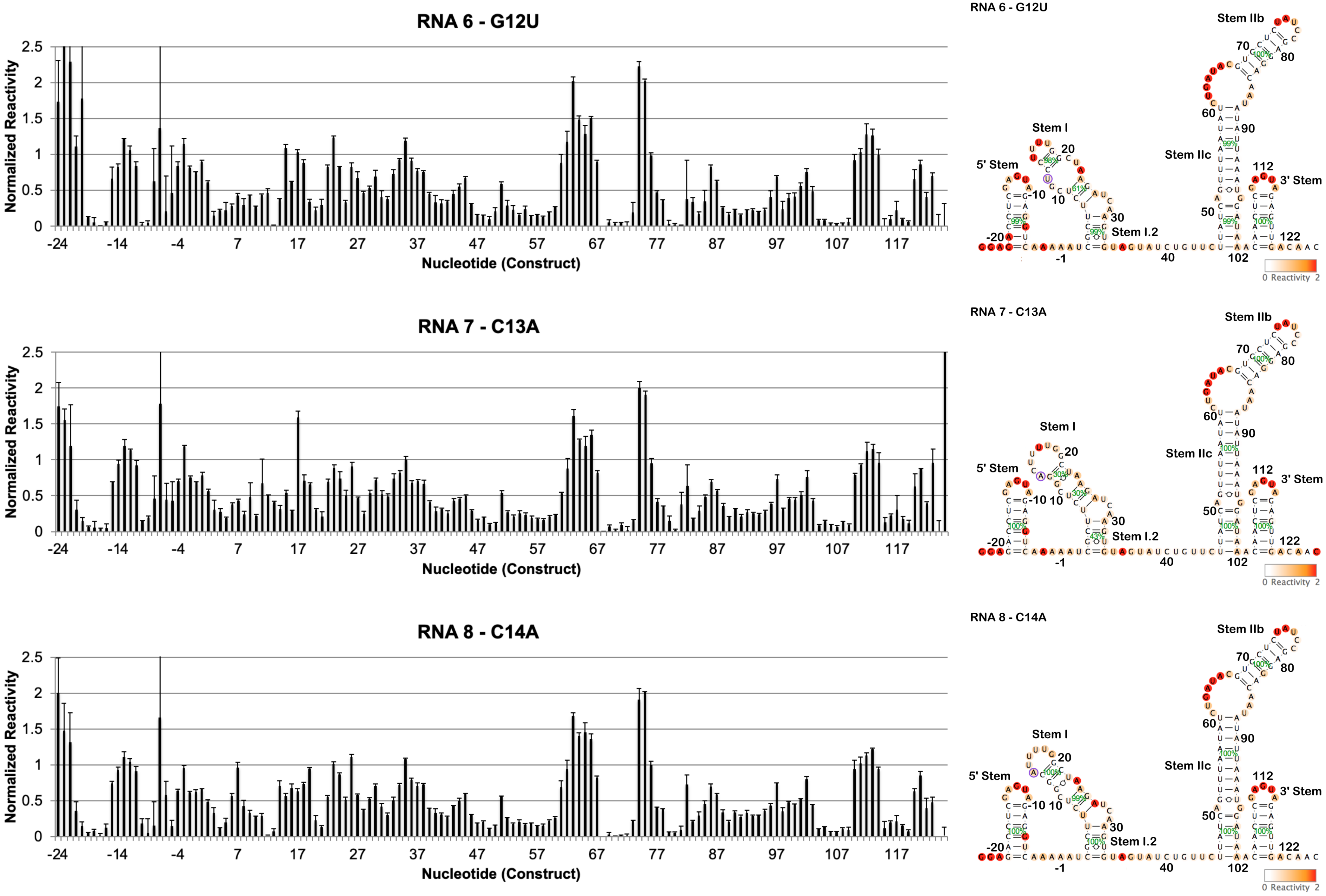

Supporting Information S2 Fig (cont)

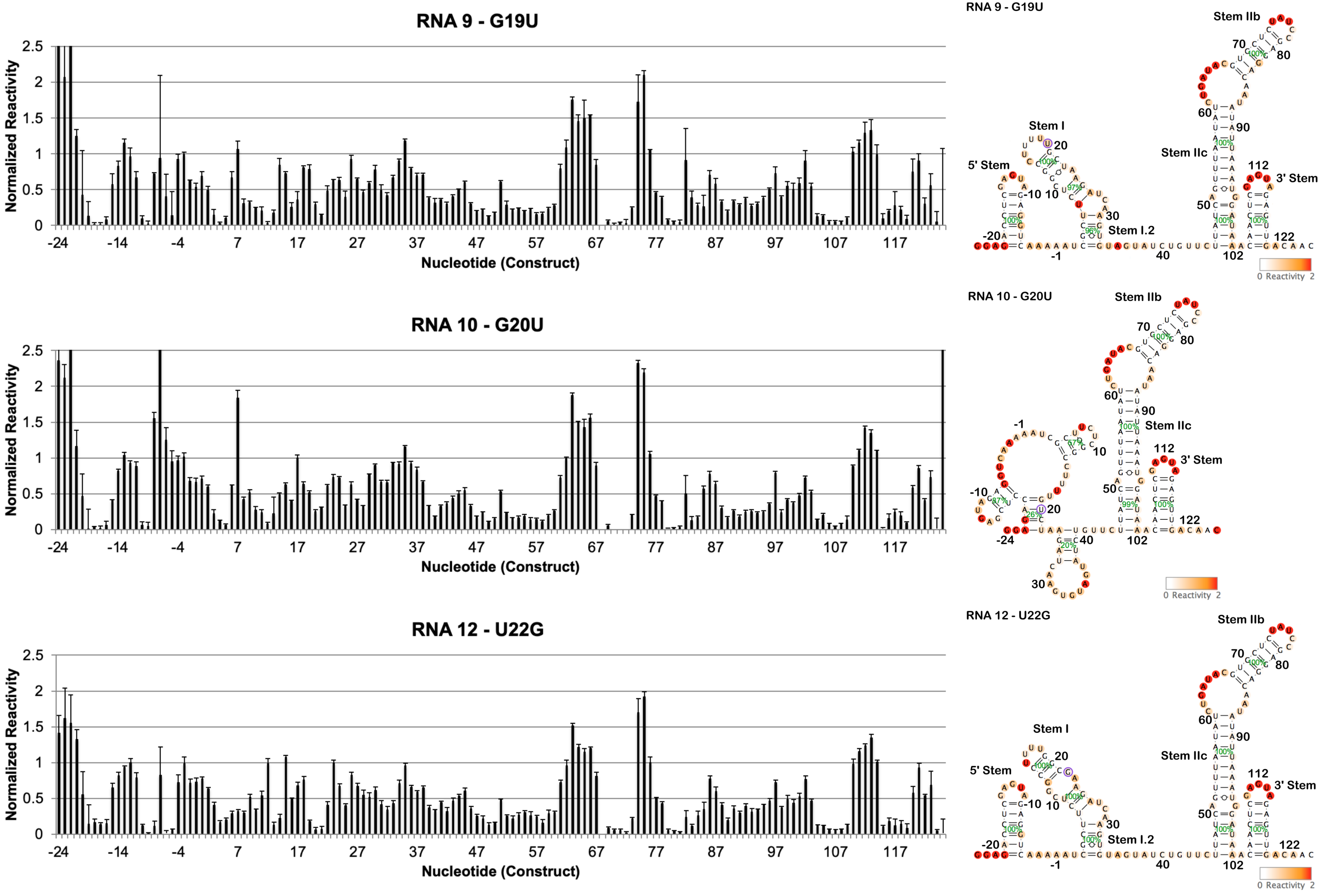

Supporting Information S2 Fig (cont)

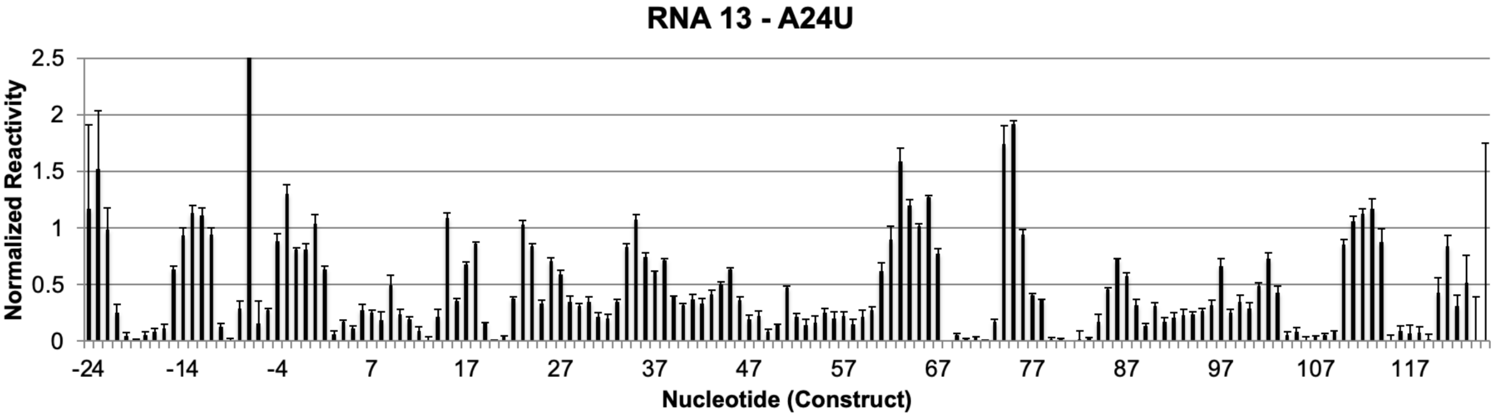

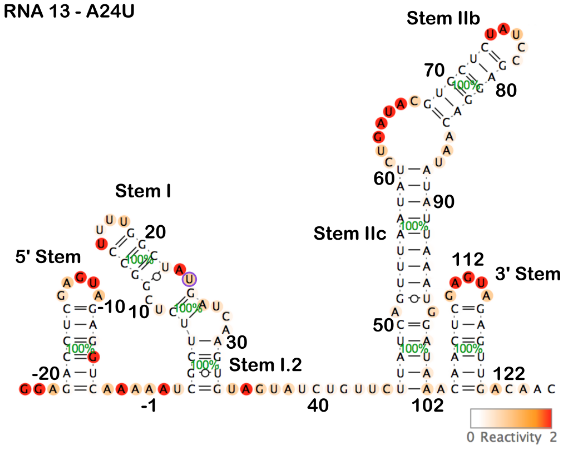

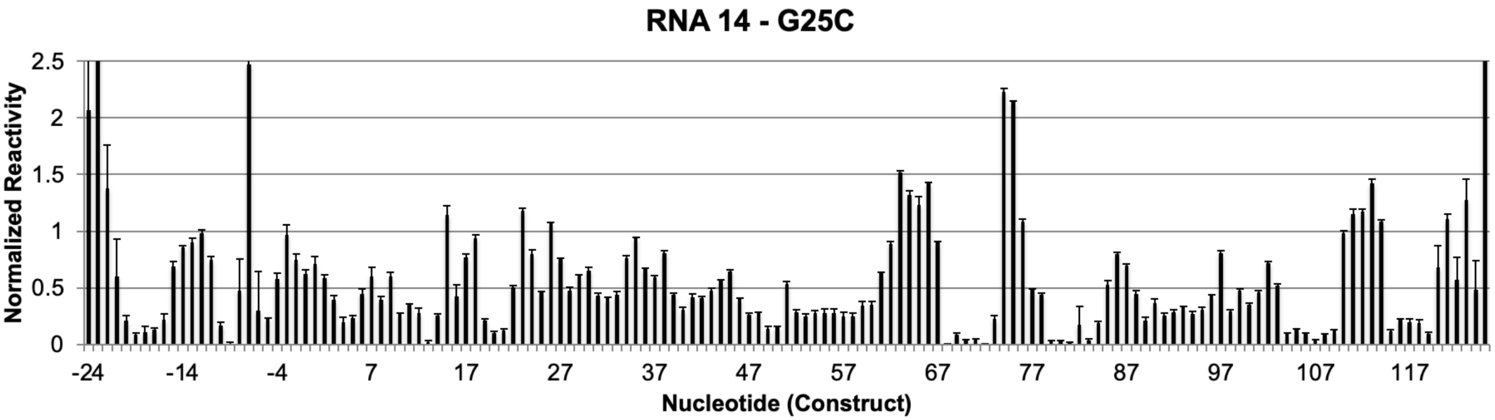

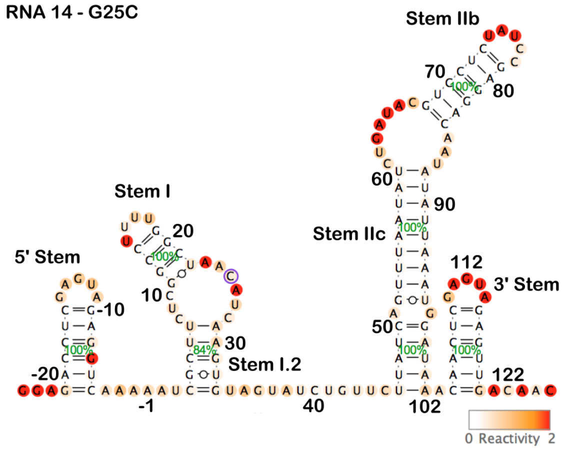

Supplement: S2 Fig — The left panels show normalized reactivities to 1m7 along with standard deviations from probing experiments conducted in triplicate or greater for the indicated synthetic U2 snRNA. The right panel shows secondary structure predictions by RNAstructure Fold based on chemical reactivity represented by the color relative to the lower right schematic. Mutations are highlighted in a purple circle. Percentages shown in green represent the probability of each structural element. (PDF) [file pone.0258551.s002.pdf]
