# Supplementary material for: U2 snRNA structure is influenced by SF3A and SF3B proteins but not by SF3B inhibitors
Source: PLoS One. 2021 Oct 14;16(10):e0258551. doi: 10.1371/journal.pone.0258551 (PMC8516221; doi:10.1371/journal.pone.0258551)

Lane legends for full gel images:

*indicates lanes that appear in the associated figure.

VU090619_Fig1 Lanes:

1. *A-complex Elution 1 (purified 8/28/19) treated with DMS
2. *A-complex Elution 1 (purified 8/28/19) no DMS (stop reaction)
3. *A-complex Elution 1 (purified 8/28/19) treated with 1m7
4. *A-complex Elution 1 (purified 8/28/19) no 1m7 (DMSO)
5. A-complex Elution 2 (purified 8/28/19) treated with DMS
6. A-complex Elution 2 (purified 8/28/19) no DMS (stop reaction)
7. A-complex Elution 2 (purified 8/28/19) treated with 1m7
8. A-complex Elution 2 (purified 8/28/19) no 1m7 (DMSO)
9. Stop reaction
10. *ddTTP (A)
11. *ddGTP (C)
12. *ddCTP (G)
13. *ddATP (U)
14. A-complex Elution 1 (purified 8/30/19) treated with DMS
15. A-complex Elution 1 (purified 8/30/19) no DMS (stop reaction)
16. A-complex Elution 1 (purified 8/30/19) treated with 1m7
17. A-complex Elution 1 (purified 8/30/19) no 1m7 (DMSO)
18. A-complex Elution 2 (purified 8/30/19) treated with DMS
19. A-complex Elution 2 (purified 8/30/19) no DMS (stop reaction)
20. A-complex Elution 2 (purified 8/30/19) treated with 1m7
21. A-complex Elution 2 (purified 8/30/19) no 1m7 (DMSO)

VU110719_Fig1 lanes

1. *ddTTP (A)
2. *ddGTP (C)
3. *ddCTP (G)
4. *ddATP (U)
5. *SSA-Inhibited-complex Elution (purified 11/05/19) no 1m7 (DMSO)
6. SSA-Inhibited-complex Elution (purified 11/05/19) treated with 1m7 (20 mM)
7. *SSA-Inhibited-complex Elution (purified 11/05/19) treated with 1m7 (10 mM)
8. SSA-Inhibited-complex Elution (purified 11/05/19) no DMS (stop reaction)
9. *SSA-Inhibited-complex Elution (purified 11/05/19) treated with DMS (1:6)
10. SSA-Inhibited-complex Elution (purified 11/05/19) treated with DMS (1:3)
11. ddTTP (A)
12. ddGTP (C)
13. ddCTP (G)
14. ddATP (U)
15. SSA-Inhibited-complex Elution (purified 11/05/19) no 1m7 (DMSO)
16. SSA-Inhibited-complex Elution (purified 11/05/19) treated with 1m7 (20 mM)
17. SSA-Inhibited-complex Elution (purified 11/05/19) treated with 1m7 (10 mM)
18. SSA-Inhibited-complex Elution (purified 11/05/19) no DMS (stop reaction)
19. SSA-Inhibited-complex Elution (purified 11/05/19) treated with DMS (1:6)
20. SSA-Inhibited-complex Elution (purified 11/05/19) treated with DMS (1:3)

VU050517_Fig2 lanes

1. U2 snRNP in HeLa nuclear extract at 150 mM KCl no DMS (stop reaction)
2. U2 snRNP in HeLa nuclear Extract at 420 mM KCl no DMS (stop reaction)
3. *ddTTP (A)
4. *ddGTP (C)
5. *U2 snRNP in HeLa nuclear extract at 150 mM KCl no DMS
6. *U2 snRNP in HeLa nuclear extract at 150 mM KCl treated with DMS
7. *U2 snRNP in HeLa nuclear extract at 420 mM KCl treated with DMS
8. *Isolated U2 snRNA treated with DMS
9. *Isolated U2 snRNA no DMS
10. *U2 snRNP in HeLa nuclear Extract at 420 mM KCl no DMS
11. *U2 snRNP in HeLa nuclear extract at 150 mM KCl no DMS
12. *U2 snRNP in HeLa nuclear extract at 150 mM KCl treated with DMS
13. *U2 snRNP in HeLa nuclear extract at 420 mM KCl treated with DMS
14. *Isolated U2 snRNA treated with DMS
15. *Isolated U2 snRNA no DMS
16. *U2 snRNP in HeLa nuclear extract at 420 mM KCl no DMS
17. *ddTTP (A)
18. *ddGTP (C)
19. U2 snRNP in HeLa nuclear extract at 150 mM KCl no DMS (stop reaction)
20. U2 snRNP in HeLa nuclear Extract at 420 mM KCl no DMS (stop reaction)

VU070617_Sup. Fig1 lanes

1. *Isolated U2 snRNA with SSA treated with DMS
2. *Isolated U2 snRNA treated with DMS
3. *U2 snRNP in HeLa nuclear extract at 420 mM KCl treated with SSA with DMS
4. *U2 snRNP in HeLa nuclear extract at 420 mM KCl treated with DMS
5. *U2 snRNP in HeLa nuclear extract at 150 mM KCl with SSA treated with DMS
6. *U2 snRNP in HeLa nuclear extract at 150 mM KCl treated with DMS
7. *ddGTP (C)
8. *ddTTP (A)
9. Isolated U2 snRNA with SSA no DMS (stop reaction)
10. *Isolated U2 snRNA no DMS (stop reaction)
11. U2 snRNP in HeLa nuclear extract at 420 mM KCl with SSA no DMS (stop reaction)
12. U2 snRNP in HeLa nuclear extract at 420 mM KCl no DMS (stop reaction)
13. U2 snRNP in HeLa nuclear extract at 150 mM KCl with SSA no DMS (stop reaction)
14. U2 snRNP in HeLa nuclear extract at 150 mM KCl no DMS (stop reaction)

VU090619_Fig1

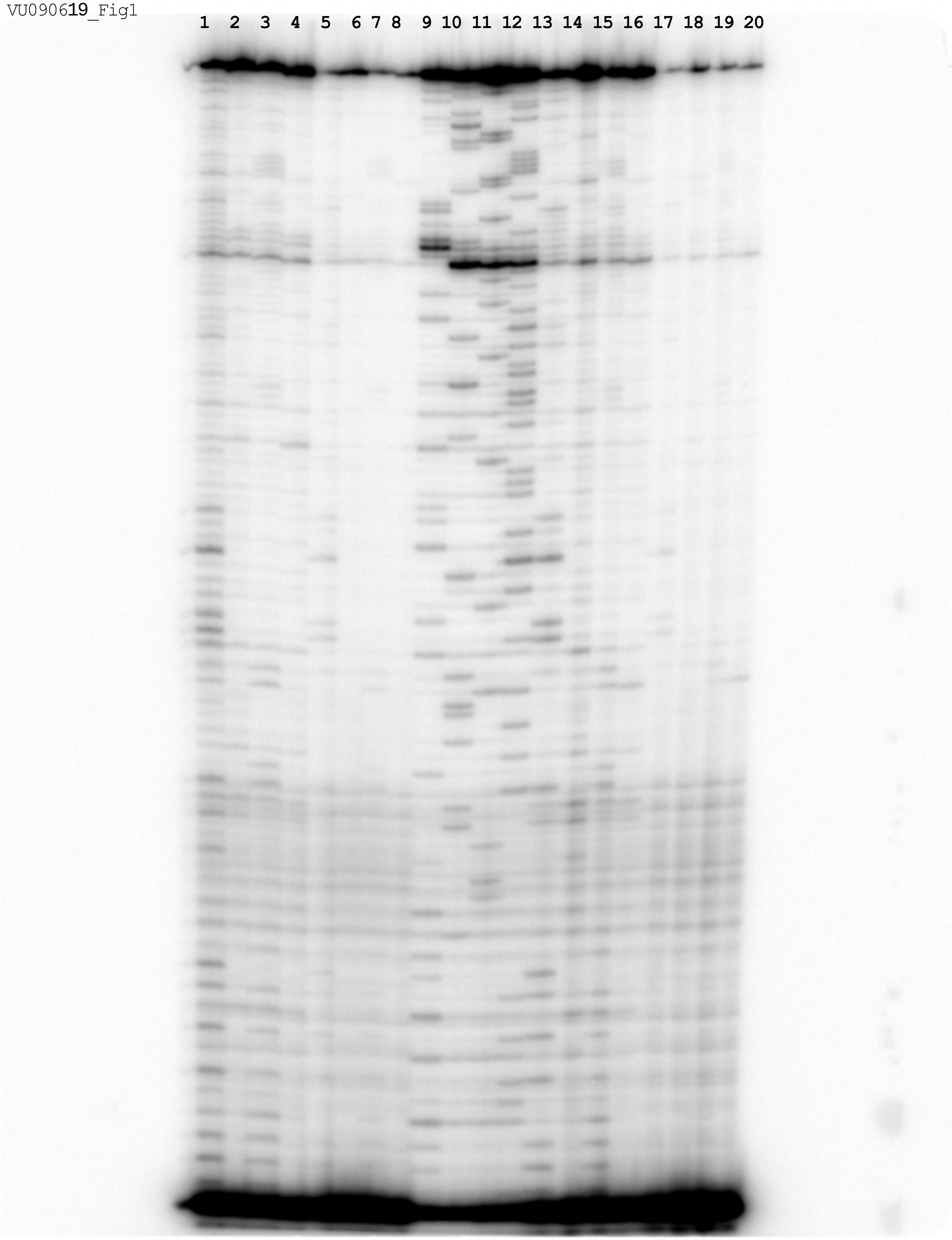

VU110719_Fig1

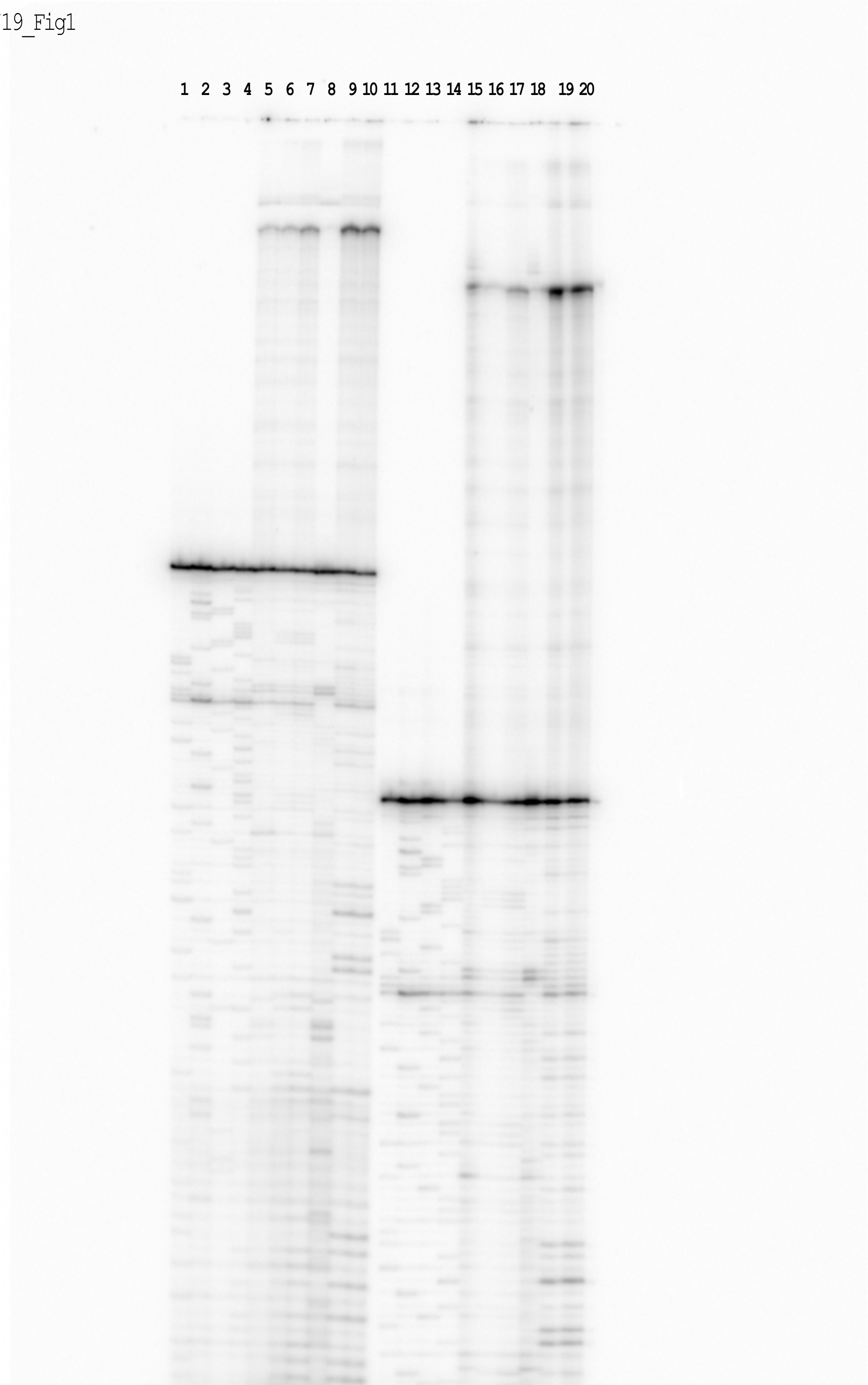

VU070617_S1 Fig.

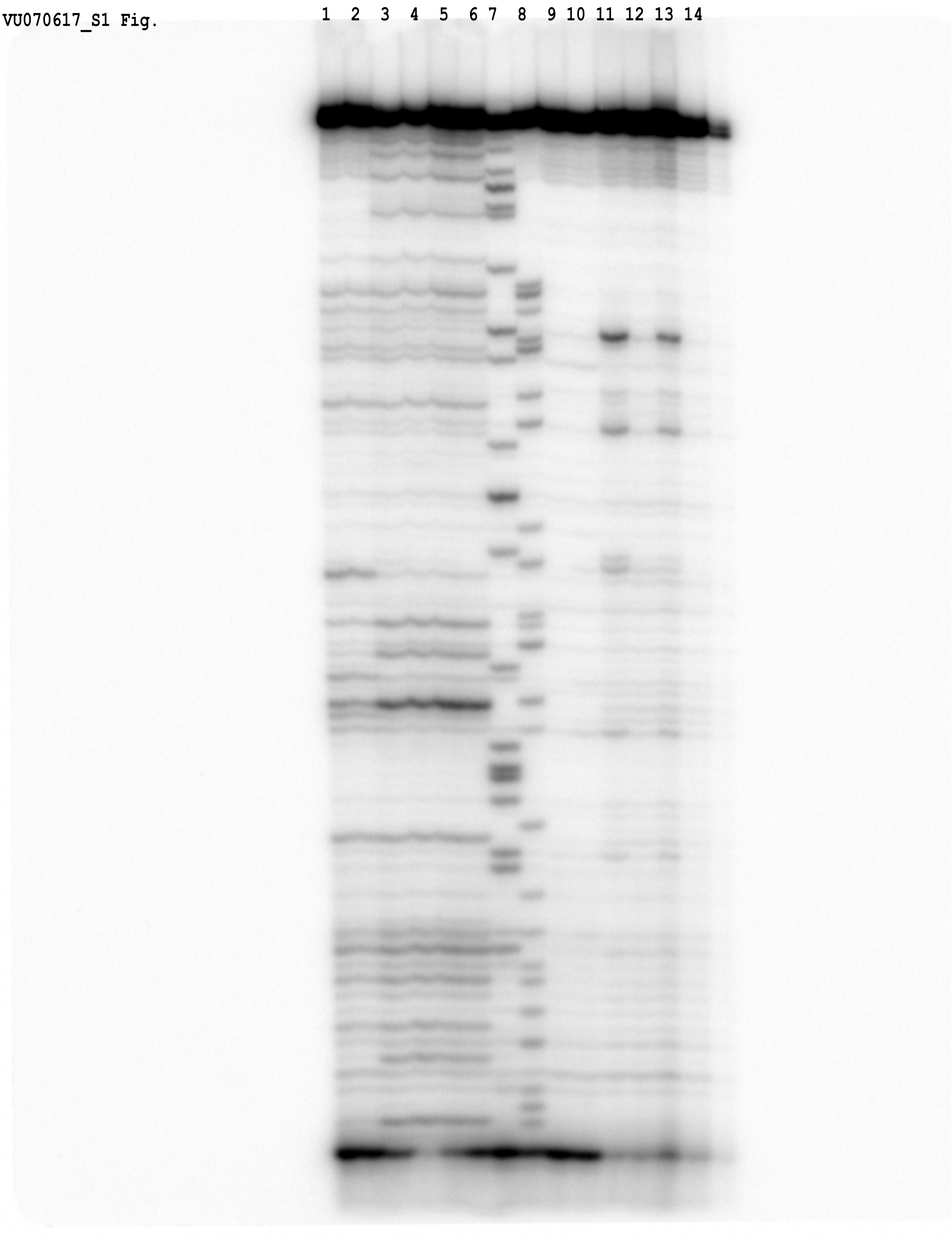

VU050527_Fig2

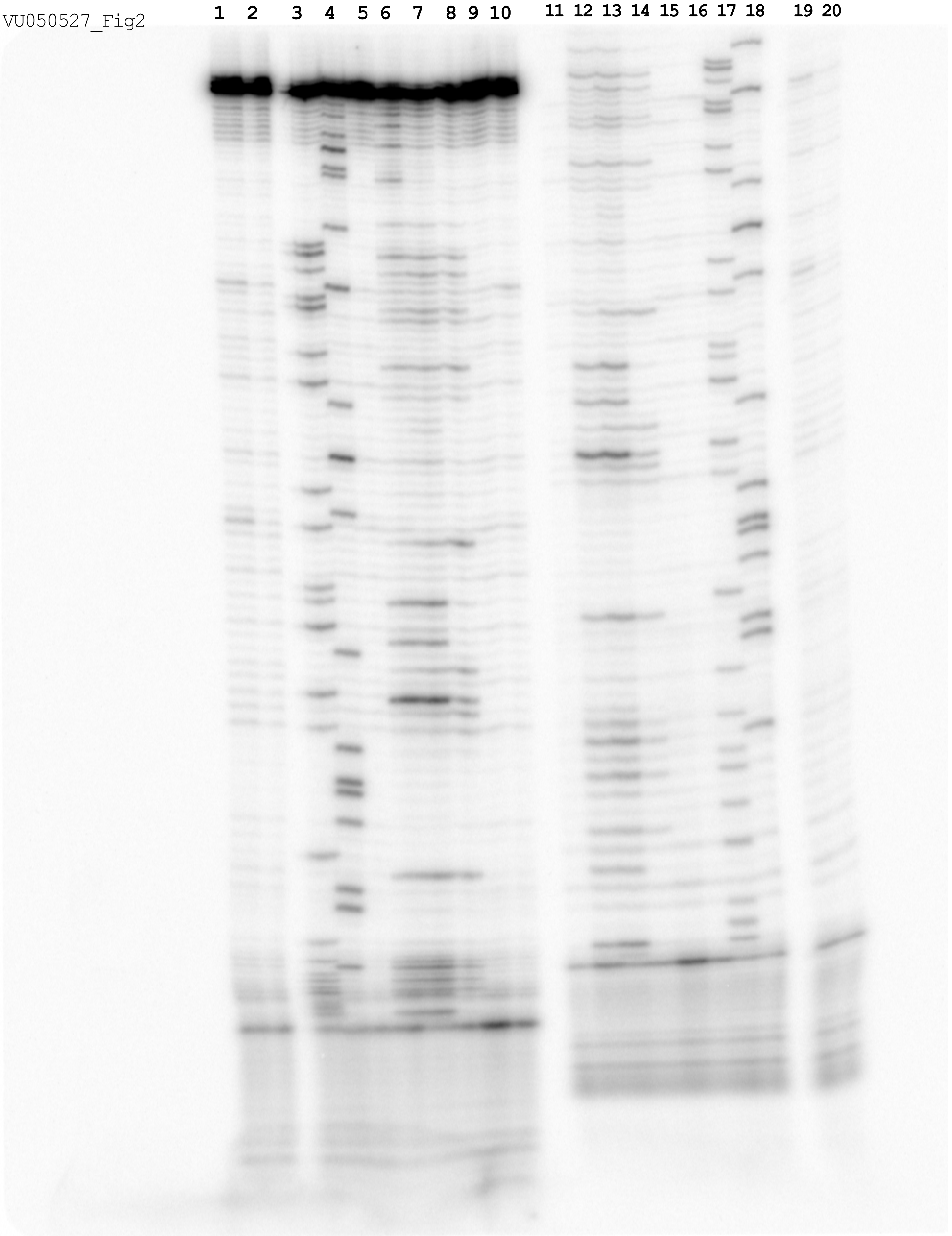

Supplement: S1 Raw images — (PDF) [file pone.0258551.s003.pdf]
